# An Equity-Embedded, Protocol-Agnostic Pre-Trial Navigation Model for Canadian Blood Cancer Trials: Findings from the Myeloma Canada Phase 0 Workshop

**DOI:** 10.3390/curroncol33070433

**Published:** 2026-07-20

**Authors:** Gabriele Colasurdo, Alvina Nadeem, Nina Mason, Juliette Royer, Stephanie Soltys, Henry Chan, Richard K. Plante, Julie Stakiw, Joseph R. Mikhael, Michelle Oana

**Affiliations:** 1 Myeloma Canada, Dorval, QC H9P 2V4, Canada; gcolasurdo@myeloma.ca; 2 Alvina N. Consulting, Montreal, QC J5Y 0J4, Canada; 3 Arthur JE Child Comprehensive Cancer Centre, Cancer Care Alberta, Calgary, AB T2N 5G2, Canada; 4 Halton-Peel Myeloma Support Group, Guelph, ON N1H 5V3, Canada; 5 Johnson & Johnson, Toronto, ON M3C 1L9, Canada; 6 Independent Researcher, Toronto, ON M3B 3B4, Canada; 7 RKP NEXUS Consulting, Toronto, ON M4G 2N5, Canada; 8 Saskatoon Cancer Centre, University of Saskatchewan, Saskatoon, SK S7N 4H4, Canada; 9 Translational Genomics Research Institute (TGen), City of Hope Comprehensive Cancer Center, Phoenix, AZ 85004, USA

**Keywords:** clinical trial navigation, health equity, human-centred design, clinical trial readiness, patient navigation, equity, diversity, and inclusion

## Abstract

**Background:** Inequitable access to clinical trials persists in blood cancers despite ongoing equity, diversity, and inclusion (EDI) efforts. Despite the critical role of clinical trials in improving survival and outcomes, recruitment remains suboptimal, limiting patient access to potentially life-saving therapies. Practical and scalable approaches are therefore needed to address the non-medical barriers that hinder patient readiness upstream of enrolment. **Methods:** Myeloma Canada led a national, multi-phase initiative using human-centred design (HCD) to operationalize EDI in clinical trials. Following an initial systems level workshop, the two-day Phase 0 workshop used a HCD approach that convened a purposively selected multidisciplinary group of stakeholders to co-design operational solutions for non-medical barriers affecting trial participation for patients. Given the use of purposive sampling, the results should be interpreted as reflecting a balanced range of diverse, informed perspectives across the Canadian clinical trial ecosystem. **Results:** Participants identified persistent cultural, logistical, financial, and linguistic barriers, along with fragmented awareness of available supports. Across diverse personas and care settings, all groups independently converged on a human-centred, equity-focused pre-trial navigation model supported by simple digital tools, including AI-enabled infrastructure drawing on curated resources from validated sources with appropriate governance, privacy, and oversight. Digital tools were proposed to support, rather than replace, human support and to align with existing health system realities. **Conclusions:** This hypothesis-generating work proposes a feasible, sustainable, and scalable equity-embedded, protocol-agnostic navigation framework. Its external hub-and-spoke structure can reduce non-medical barriers, strengthen trial access and accrual, and enhance representativeness. Pilot implementation that assesses feasibility, uptake, workflow impact, equity effects, and implementation burden is warranted.

## Simple Summary

Clinical trials are essential for advancing innovative healthcare, play a critical role in improving patient outcomes, and can provide life-changing treatment options, particularly in cancer and blood cancers. Yet many patients cannot take part because of surmountable challenges such as travel, cost, demanding work schedules, language/cultural barriers, and fragmented support. These barriers limit participation, reduce the diversity of trial populations, and affect Canada’s ability to support timely recruitment and access to diverse patient populations in an increasingly competitive global clinical trial environment. To better understand how to remove these obstacles, Myeloma Canada brought together patients, clinicians, researchers, policy partners, and industry stakeholders for a national workshop. Using fictitious patient scenarios that reflected common challenges, participants co-designed a model for a pre-trial navigation service focused on addressing practical needs early in the process. The approach relies on trained navigators, supported by simple digital tools drawing on curated, validated information to help patients considering a trial coordinate logistical requirements and access available resources. This work highlights a feasible, scalable strategy to improve fairness in trial access and strengthen Canada’s clinical trial readiness.

## 1. Introduction

Clinical trials play an increasingly integral role across the cancer treatment continuum. In addition to advancing knowledge and improving outcomes, they often provide patients with access to promising novel therapies that may be otherwise unavailable through standard care. For some patients, participation in a clinical trial may represent the most appropriate therapeutic option at a given point in their disease course. Despite this, access to clinical trials remains inequitable, creating gaps in evidence for diverse populations and limiting opportunities for underrepresented groups to benefit from emerging therapies.

Within this evolving research landscape, Canada is emerging as a strategically positioned clinical trial hub, supported by its universal healthcare system and diverse population representing more than 450 ethnic and cultural origins [1,2,3]. However, Canada continues to face challenges in meeting recruitment targets and sustaining timely trial activation, particularly in the context of increasing international competition for oncology studies, underscoring the need for more efficient and equitable trial pathways. Global pressures on trial activation and accrual are intensifying, driven by regulatory pressures, rising costs, geopolitical uncertainty, and expectations for more representative trial populations [1,2,4]. By strengthening patient readiness and addressing upstream non-medical barriers that affect feasibility, timelines, and accrual efficiency, this initiative may also enhance Canada’s attractiveness as a setting for high-quality clinical trials.

Operationalizing equity, diversity, and inclusion (EDI) within clinical trials requires practical, sustainable approaches that embed patient-centred practices upstream of enrolment. EDI refers to deliberate efforts to ensure that clinical trial populations reflect the diversity of patients affected by disease, and that trial processes enable fair access, meaningful participation, and the generation of representative, generalizable data. This includes supporting patients and care teams before trial participation is considered. In this context, non-medical barriers refer to the practical, structural, and contextual factors that drive many EDI-related disparities in trial access, such as logistical burden, financial strain, language barriers, sociocultural factors, and medical mistrust [5,6].

Although several national EDI initiatives and frameworks exist within the clinical trial ecosystem, many remain underutilized due to fragmented access, limited awareness, and poor integration into clinical workflows [7]. These challenges reflect deeper gaps in how trials are communicated, implemented, and accrued, not simply logistical constraints [7]. As a result, clinical trial participation has become more complex and burdensome for patients.

To better understand and address these persistent challenges, Myeloma Canada convened the Phase 0 Workshop to design an EDI-embedded, patient-centred model for improving trial readiness in blood cancers. The goal was to identify practical, scalable strategies that can enhance accrual, strengthen representativeness, and reduce non-medical barriers without requiring protocol or site-level modifications.

While several patient navigation and EDI initiatives exist within oncology, many are embedded within specific institutions, disease programs, or clinical trial processes. The Phase 0 model differs by focusing on upstream trial readiness through a protocol-agnostic navigation structure designed to operate across multiple trials and care settings.

## 2. Materials and Methods

The initiative began with the Health eMatters IMPACT Workshop, held in April 2024. This national, multi-stakeholder event used a human-centred design (HCD) approach and case-based exercises to explore barriers to clinical trial accrual and identify upstream, patient-centred solutions. The IMPACT workshop explored a broad spectrum of non-medical barriers including logistical challenges as well as cultural, psychosocial, and stigma-related influences through structured empathy and barrier-identification exercises to highlight diverse patient needs. Personas used during IMPACT did not represent real individuals and were developed to surface these non-medical barriers in a user-centred way.

Building on these insights, Myeloma Canada convened the Phase 0 Workshop in March 2025 with an added focus on practical, structural, and contextual barriers that could be addressed through a navigation-based service model. Workshop participants were purposively selected by Myeloma Canada to reflect a broad cross-section of the Canadian clinical trial ecosystem, including patients and caregivers, patient advocacy groups, hematologists, clinical research nurses and coordinators, Indigenous patient navigators, drug access navigators, social workers, clinical research organization representatives, trial sponsors, policy stakeholders, ethics board members, and government representatives. Participants also reflected diverse geographies and care settings, from large academic cancer centres to smaller regional hospitals and remote communities. During workshop design, Myeloma Canada also aimed to balance professional roles within each group (e.g., avoiding over-representation of any single stakeholder type and mixing participants who did not routinely work together) to help reduce dominance by particular perspectives and support more balanced discussion. Given the use of purposive sampling, this approach enabled inclusion of diverse stakeholder perspectives but may have also introduced selection bias; therefore, findings should be interpreted as reflecting a diverse range of informed perspectives across the Canadian clinical trial ecosystem rather than a statistically representative sample.

Day 1 focused on a collective intake process delivered through plenary presentations and panel discussions, where participants examined the real-world impact of non-medical barriers and reviewed analogous navigation and support models including patient support programs, drug access navigation, social work practices, and emerging AI-enabled tools to ground the co-design work in diverse operational and patient-centred perspectives.

**Figure A1:**
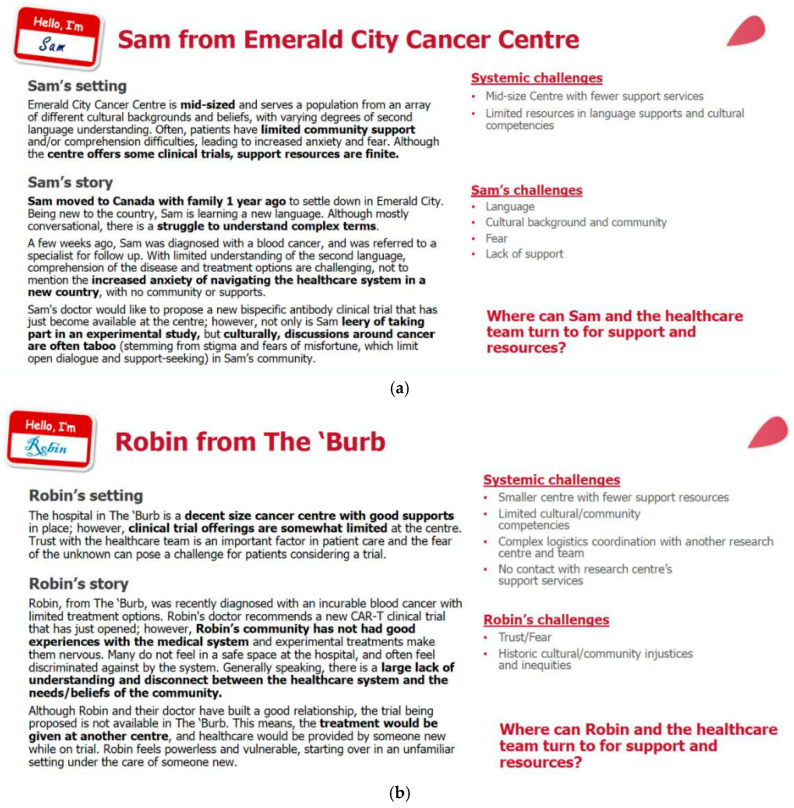

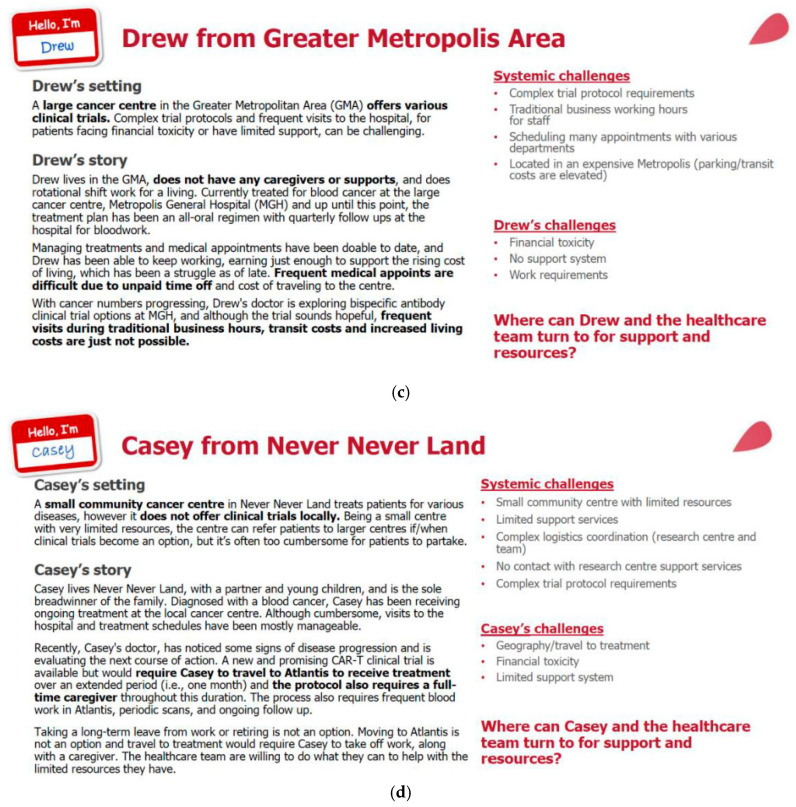
Blood cancer-specific personas: (**a**) Sam from Emerald City; (**b**) Robin from the ‘Burb; (**c**) Drew from Greater Metropolis Area; (**d**) Casey from Never Never Land.

Day 2 followed a structured design sprint format. Twelve teams worked with four fictitious blood cancer-specific personas (Appendix A, Figure A1), which were derived from the original IMPACT personas. Three teams were assigned to each persona to test whether different groups, starting from the same patient and care setting, would generate convergent or divergent solutions. Unlike the IMPACT workshop, where personas were disease-agnostic and situated in generic care settings, the Phase 0 workshop used blood-cancer-specific personas grounded in trial-related logistical, financial, cultural, and systemic realities. These refined Phase 0 personas retained the same core non-medical barriers but added blood-cancer-specific clinical context and nuance across care settings, which better reflected upstream trial-related challenges and enabled teams to stress-test solutions across different clinical environments.

**Figure 1:**
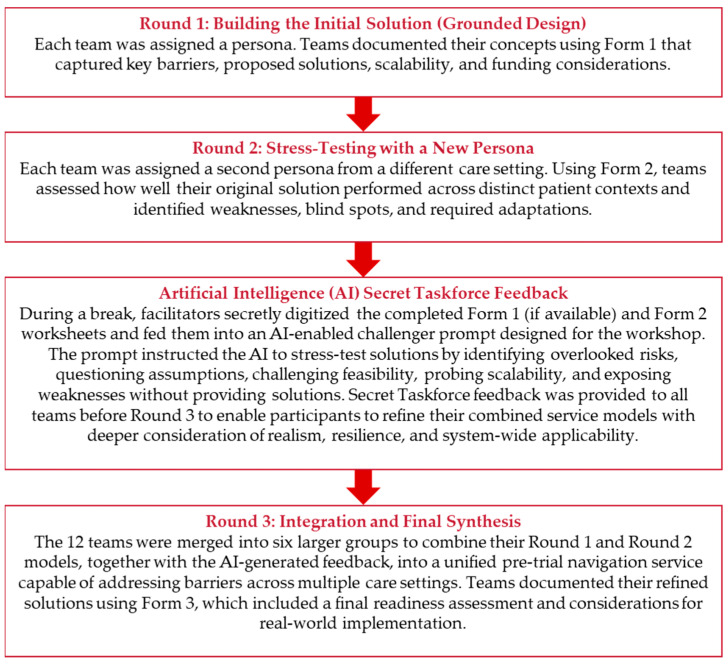
Structured Design-sprint Format Used on Day 2.

**Figure A2:**
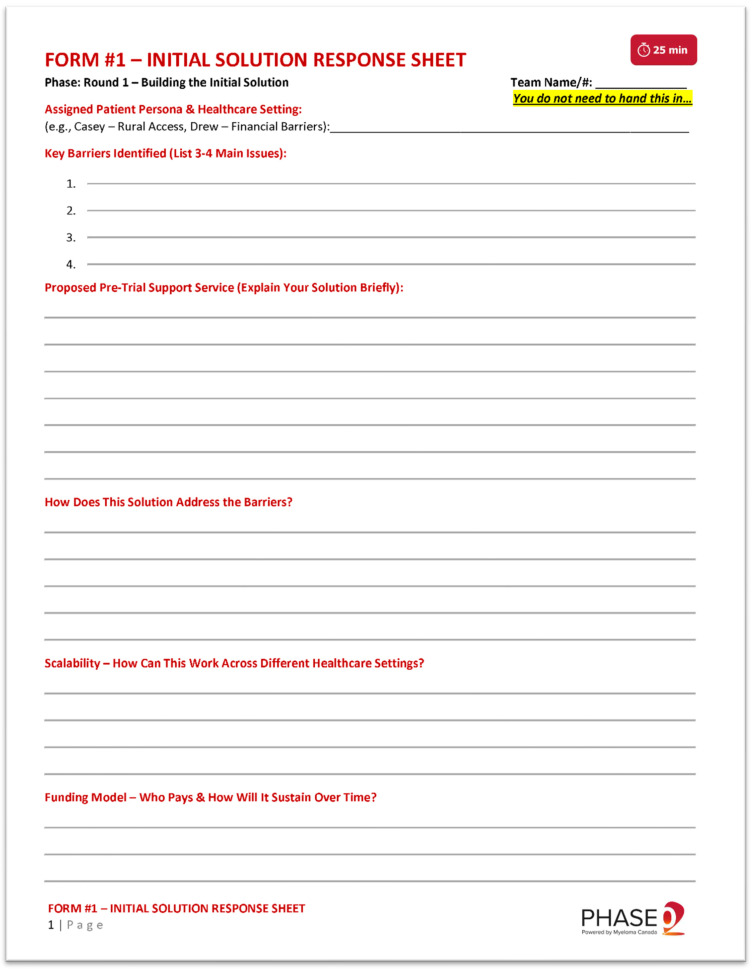
Form 1.

**Figure A3:**
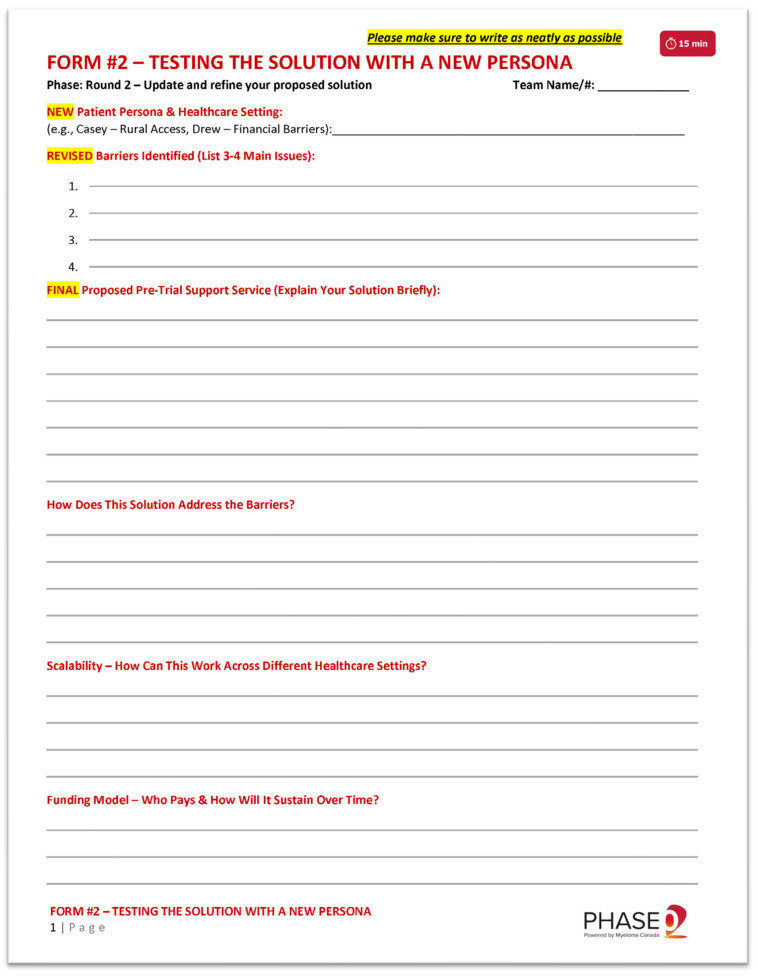
Form 2.

**Figure A4:**
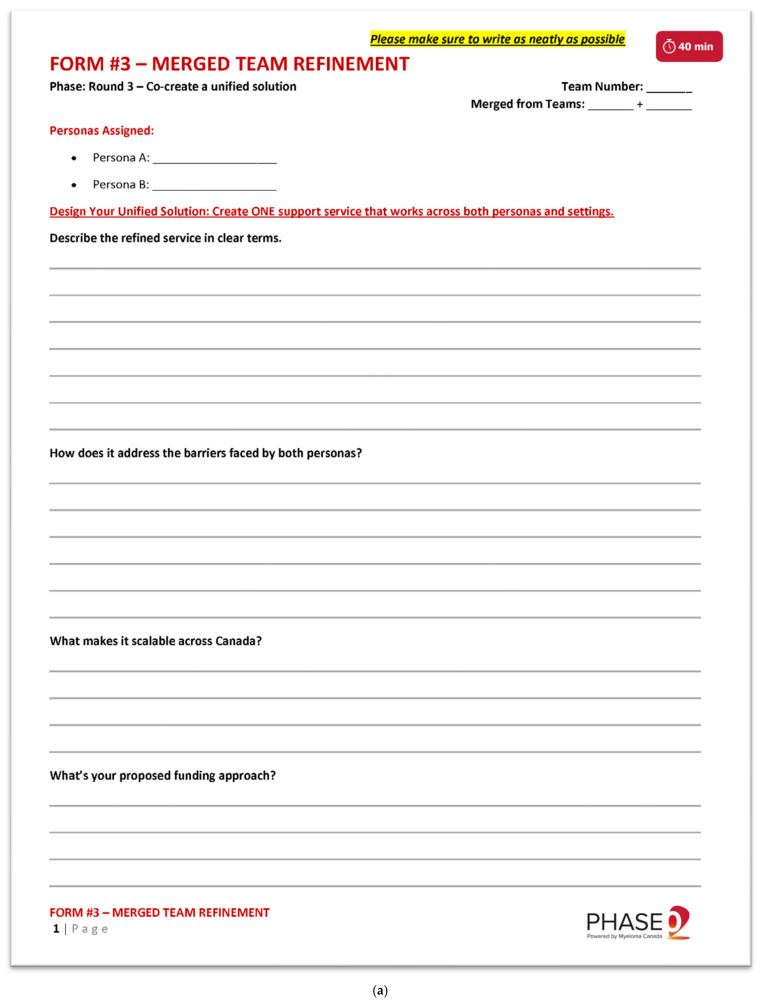

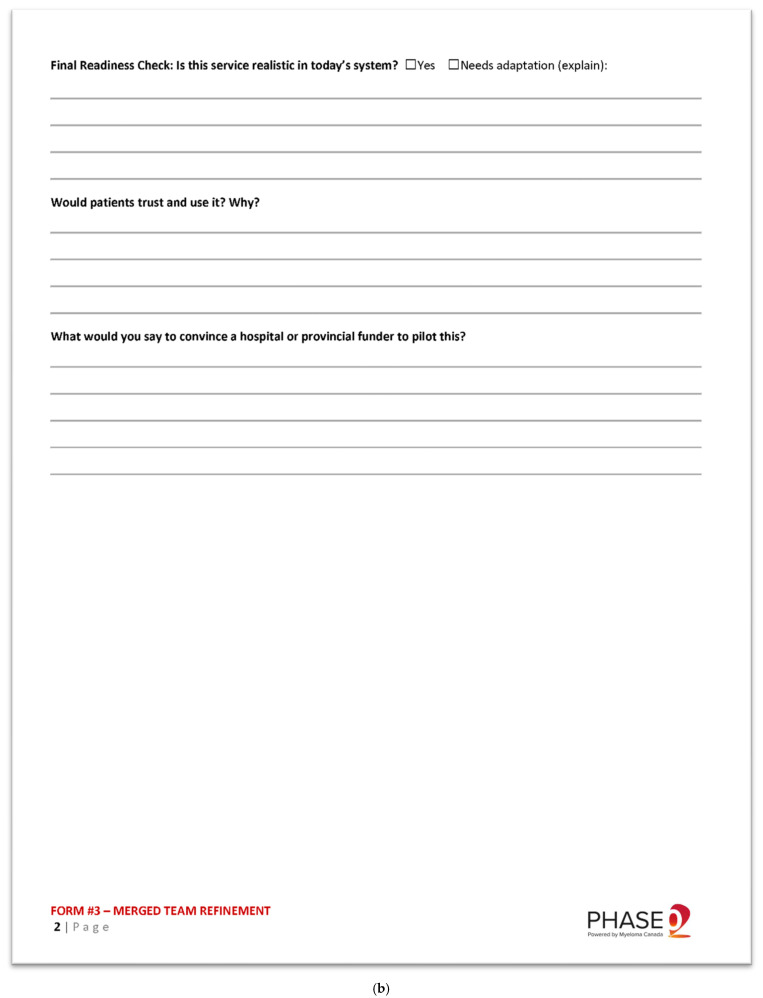
Form 3: (**a**) Page 1; (**b**) Page 2.

Using an iterative HCD methodology, teams progressed through three rounds of co-design and documented their concepts, stress tests, and refinements using Forms 1–3 (Appendix A, Figure A2, Figure A3 and Figure A4), while designing a pre-trial navigation service that operated within existing trial protocols and site infrastructure (Figure 1). Workshop outputs (Forms 1–3) were reviewed descriptively to identify recurring non-medical barriers, common service components, and areas of convergence across personas and care settings, rather than to generate quantitative measures of agreement. At the end of Day 2, all six teams presented their unified models. Participants then completed an anonymous QR-based vote, supplemented by a sticker selection system, to identify the most scalable and impactful solution. Consensus on the final pre-trial navigation model was achieved through this iterative synthesis and voting process, with the consolidated model reflecting elements that recurred across teams and were most frequently selected as feasible and impactful.

## 3. Results

The core components of the consolidated pre-trial navigation model are summarized in Table 1.

### 3.1. Barriers Identified

Participants identified persistent non-medical barriers affecting trial readiness, including relational trauma and medical mistrust, logistical and financial burden, linguistic challenges, and fragmented access to available supports. These barriers were noted across both urban and rural settings and were especially pronounced for individuals living remotely or working in unstable employment conditions. Participants agreed that a central coordinating mechanism is needed to consolidate existing resources and support navigation across diverse patient circumstances. These themes recurred across multiple teams and personas, suggesting broad agreement regarding the key non-medical barriers affecting trial readiness. Participants emphasized that these barriers affect not only patients but also caregivers, who often shoulder additional logistical, financial, and emotional burdens that can influence trial readiness and participation.

### 3.2. Convergence on a Pre-Trial Navigation Model

Across all working groups, and despite starting from different personas and care settings, participants independently converged on a common model, reflecting shared recognition of recurring non-medical barriers across real-world trial pathways. This convergence on a highly similar navigation concept across twelve teams suggests a strong degree of consensus regarding the need for a coordinated, pre-trial navigation service. Each group proposed a human-centred, equity-focused pre-trial navigation service delivered by external navigators and supported by digital tools, including AI-enabled infrastructure with appropriate governance, privacy, and oversight.

### 3.3. Core Components of the Proposed Model

Groups developed remarkably similar service designs, coalescing around a small number of core components that together define the proposed pre-trial navigation model.

#### 3.3.1. Human Navigation

Professionals (e.g., nurses, navigators, peer guides, or social workers) would serve as trusted points of reference to identify individual non-medical barriers and coordinate available supports such as transportation, lodging, language support (including translation), cultural mediation, financial assistance, and engagement with patient partners.

#### 3.3.2. Technology-Enabled Supports for Navigator-Use Only

To support consistent delivery of the navigation service across settings, teams identified a limited set of enabling digital supports intended to assist navigators rather than replace human interaction. Digital tools including resource libraries, scheduling and reminder systems, checklists, and AI-assisted workflows were proposed to support navigators, enhance coordination, and maintain consistency. AI-enabled infrastructure emerged during workshop discussions as one of several potential enablers of navigation, primarily in relation to information organization, scheduling, and workflow support. While not all teams emphasized AI to the same extent, participants agreed that any digital tools should be navigator-facing, and implemented with appropriate governance, privacy safeguards, and oversight. Teams emphasized the importance of offering low-complexity technology formats where needed.

#### 3.3.3. Equitable Access Considerations

Teams highlighted the need for multiple modes of engagement, such as phone-based navigation and printable materials, to ensure accessibility and prevent digital inequities.

#### 3.3.4. Scalability

A hub-and-spoke structure was identified as the most feasible delivery model, enabling consistent service across regions without requiring trial protocol or site changes. Scalability strategies included training existing professionals both to recognize when to engage in the service and, where appropriate, to serve as trained pre-trial navigators for the proposed service.

#### 3.3.5. Funding and Sustainability

Teams proposed blended funding models, with appropriate governance structures, involving sponsors, provincial health authorities, non-profits, and foundations to support an external service independent of hospital operating budgets.

## 4. Discussion

National and industry reports identify Canada as well-positioned for high-quality clinical research, yet persistent non-medical barriers continue to limit participation and threaten representativeness [1,2]. These barriers, including mistrust, logistical and financial burdens, language challenges, and fragmented support, remain prevalent among patients with blood cancers, despite longstanding recognition in the literature [5,6]. Their persistence underscores that policy intent alone has not translated into system-level change.

By addressing non-medical barriers upstream, this navigation strategy may work synergistically with advances in blood cancer therapeutics, potentially helping clinical innovation translate more consistently into real-world patient benefit; however, these anticipated benefits will require prospective evaluation.

Workshop participants consistently highlighted the absence of coordinated patient-centred navigation as a key upstream gap in trial readiness. Because these barriers emerge early and vary widely, protocol-level or site-specific adjustments are insufficient. Evidence demonstrates that navigation can improve trial engagement, awareness, and enrolment among underserved populations [8], supporting an external, centralized service model that enhances coordination without adding workload within clinical environments. Implementation of a blended funding model will require clear governance to ensure neutrality and transparency while avoiding undue influence. It must also align with institutional policies, ethics oversight, and regulatory compliance requirements.

The proposed model also incorporates digital tools to support navigators. This reflects broader calls for equity-informed digital strategies in clinical research and acknowledges that poorly designed digital solutions can exacerbate disparities, particularly among individuals with limited digital access or literacy [6]. By pairing human navigation with enabling technology, rather than replacing interpersonal support, the model is designed to improve coordination while maintaining accessibility. Any future digital implementation would need to draw on curated resources from validated sources with appropriate data governance, privacy safeguards, and oversight. At this stage, AI-enabled tools are proposed as optional, navigator-facing supports to organize information and workflows; their clinical and operational impact remains to be established through future implementation and evaluation.

This approach is complementary to broader regulatory efforts, including increasing support for decentralized and hybrid trial models intended to expand accessibility; however, it addresses a distinct upstream need by supporting patients in navigating non-medical barriers regardless of trial design. While trial eligibility and patient selection remain inherently protocol-driven, the proposed model operates upstream by addressing non-medical barriers that influence whether patients can realistically engage with trial pathways, thereby functioning alongside, rather than within, protocol- and site-specific processes.

Taken together, these findings suggest that participants perceived a shared system-level gap in trial readiness that extends beyond individual sites, protocols, or technologies. The strong convergence across diverse workshop stakeholders suggests that this approach reflects system-level needs rather than isolated perspectives. It is also aligned with national priorities emphasizing improved efficiency, stronger representativeness, and reduced fragmentation to sustain Canada’s competitiveness in an evolving global clinical trial environment [1,2,7,9].

Moreover, the model complements existing Canadian EDI initiatives [10,11,12] by providing a practical mechanism to operationalize these frameworks in real-world trial pathways. Our findings are broadly consistent with prior evidence demonstrating that patient navigation can improve trial engagement, awareness, and enrolment among underserved populations. AI-enabled matching platforms and patient-facing navigation services primarily focus on identifying eligible trials and connecting patients to study opportunities. In contrast, the Phase 0 model targets upstream, non-medical barriers that affect a patient’s ability to become trial-ready in the first place. By addressing logistical, financial, and coordination challenges prior to formal screening and matching, the model functions as an enabling layer that may enhance the effectiveness of existing initiatives.

Future pilot implementation should incorporate predefined evaluation metrics to assess both patient- and system-level impact. Few published models address non-medical barriers through an upstream, external navigation structure that operates alongside hospital and trial workflows, making this a distinct contribution to Canadian clinical trial modernization. Given the external and protocol-agnostic nature of the model, direct linkage between navigation activity and trial enrolment is unlikely to be reliably captured due to privacy considerations, so proxy indicators will be needed as indirect measures of impact. Relevant service and process metrics may include navigation request volume, time from request to completion, types of non-medical barriers addressed, and user-reported stage of recruitment (e.g., whether a trial opportunity was identified or trial information, including informed consent materials, was provided). User-reported satisfaction with navigation support will also be important to capture experience. Additional system-level indicators, such as recruitment efficiency and site workload implications, will help assess the operational value and scalability of the model. Future work should also evaluate feasibility, stakeholder uptake, and real-world impact across diverse settings, and determine whether integrated navigation can meaningfully reduce participation inequities for patients.

### Limitations

This work has several limitations. The findings reflect the perspectives of participants from a purposively sampled national workshop and may not capture all operational realities across Canada. Although participants represented diverse roles, geographies, and care settings, selection bias and the potential influence of more vocal or experienced participants are inherent to workshop-based consensus methods. In addition, the proposed model has not yet been tested in practice, and its feasibility, workload implications, and equity impact remain to be evaluated. While the Phase 0 workshop used blood-cancer-specific scenarios to ground discussions, the resulting navigation model is intended to be disease-agnostic, as it focuses on upstream non-medical barriers that are common across diverse trial contexts. These limitations underscore that the model reflects expert consensus and highlight the need for prospective pilot implementation to assess how it functions within real-world clinical environments.

## 5. Conclusions and Future Directions

The Phase 0 workshop produced a pragmatic, equity-embedded pre-trial navigation model that integrates dedicated human-centred navigation roles with digital tools designed to support, not replace, human interaction. By centralizing navigation, tailoring support to individual patient needs, and coordinating existing services through a scalable and sustainable framework, the model offers a feasible approach to addressing non-medical barriers. As a workshop-based, hypothesis-generating initiative, this model reflects expert consensus and will require prospective pilot implementation and evaluation before its feasibility, operational impact, and equity effects can be confirmed. Although broader system changes remain necessary, the service is intentionally protocol-agnostic and can function regardless of future shifts in trial design or infrastructure.

By operating alongside existing care structures rather than within them, the model is intended to enhance trial readiness without increasing institutional workload. Moreover, the model complements both existing and new initiatives addressing trial equity and recruitment, offering a practical coordination layer rather than a competing framework. Future work should prioritize stakeholder engagement, operational readiness, and the development of enabling digital tools for navigators. Pilot testing in selected settings will be essential to assess feasibility, adaptability, and value across diverse healthcare contexts.

## Figures and Tables

**Table 1:** Core Components of the Proposed Pre-Trial Navigation Model.

Component	Key Elements	Primary Function
Human navigation	External nurses, navigators, peer guides, or social workers	Identify non-medical barriers; Coordinate support (transportation, translation, lodging, and financial aid)
Technology supports (navigator-use only)	Resource libraries; Checklists; AI-supported scheduling and reminders	Improve coordination, efficiency, and consistency; Integrate fragmented services; Ensure low-complexity access options
Equitable access	Phone/print options; Equity-informed design	Maintain accessibility across diverse populations and prevent disparities
Scalability	Training existing professionals to recognize and refer; Hub-and-spoke architecture with regional flexibility	Provide national coverage without protocol or site modifications
Funding	Blended funding model including trial sponsors, provincial health authorities, non-profits, and foundations	Sustain a service independent of hospital budgets

## Data Availability

The data presented in this study are available in this article.
